# The levels of trypsinogen isoenzymes in ovarian tumour cyst fluids are associated with promatrix metalloproteinase-9 but not promatrix metalloproteinase-2 activation

**DOI:** 10.1054/bjoc.2001.1806

**Published:** 2001-05

**Authors:** A Paju, T Sorsa, T Tervahartiala, E Koivunen, C Haglund, A Leminen, T Wahlström, T Salo, U-H Stenman

**Affiliations:** Departments of Clinical Chemistry andObstetrics and Gynecology, Helsinki University Central Hospital, Haartmaninkatu 2, FIN-00290 Helsinki, Finland; Institute of Dentistry, University of Helsinki, Mannerheimintie 172, FIN-00140, Helsinki, Finland; Department of Biochemistry, University of Helsinki, Viikinkaari 5 D, FIN-00140, Helsinki, Finland; Department of Surgery, Helsinki University Central Hospital, Haartmaninkatu 4, FIN-00290 Helsinki, Finland; Department of Pathology, Haartman Institute, Helsinki University Central Hospital, Haartmaninkatu 4, FIN-00290 Helsinki, Finland; Department of Oral Diagnostics and Pathology, Institute of Dentistry, University of Oulu, FIN-90014, Oulu, Finland

**Keywords:** trypsin, TATI, MMP-2, MMP-9, ovarian cancer, cyst fluid

## Abstract

Proteolysis mediated by matrix metalloproteinases (MMPs) and serine proteinases is associated with cancer invasion and metastasis. Activation of latent proMMPs, and especially the proforms of the type IV collagen degrading gelatinases A and B (proMMP-2 and proMMP-9), is thought to be a critical step in this process. We have recently found that human tumour-associated trypsin-2 is a potent activator of proMMP-9 and it also activates proMMP-2 in vitro. Trypsinogen, MMP-2, and MMP-9 are expressed in ovarian cancer. To elucidate the function of trypsin in vivo, we studied whether high concentrations of trypsinogen-1, trypsinogen-2, their α_1_-proteinase inhibitor (API) complexes, and tumour-associated trypsin inhibitor (TATI) are associated with proMMP-2 and proMMP-9 activation in ovarian tumour cyst fluids. Zymography and immunofluorometric analysis of 61 cyst fluids showed a significant association between high trypsin concentrations and the activation of MMP-9 (*P*= 0.003–0.05). In contrast, the trypsin concentrations were inversely associated with the activation of MMP-2 (*P*= 0.01–0.02). Immunohistochemical analysis of ovarian tumour tissue demonstrated expression of trypsinogen-2 and TATI in the secretory epithelium. MMP-2 was detected both in stromal and epithelial cells whereas MMP-9 was detected in neutrophils and macrophage-like cells in stromal and epithelial areas. These results suggest that trypsin may play a role in the regulation of the MMP-dependent proteolysis associated with invasion and metastasis of ovarian cancer. © 2001 Cancer Research Campaign www.bjcancer.com


British Journal of Cancer (2001) **84**(10), 1363–1371
© 2001 Cancer Research Campaign
doi: 10.1054/ bjoc.2001.1806, available online at http://www.idealibrary.com on IDEAL®

http://www.bjcancer.com

### The levels of trypsinogen isoenzymes in ovarian tumour cyst fluids are associated with promatrix metalloproteinase-9 but not promatrix metalloproteinase-2 activation

**A Paju[1], T Sorsa[2], T Tervahartiala[2], E Koivunen[3], C Haglund[4], A Leminen[5], T Wahlström[6], T Salo[7] and U-H Stenman[1]**

Departments of [1]Clinical Chemistry and [5]Obstetrics and Gynecology, Helsinki University Central Hospital, Haartmaninkatu 2, FIN-00290 Helsinki, Finland; [2]Institute of Dentistry, University of Helsinki, Mannerheimintie 172, FIN-00140, Helsinki, Finland; [3]Department of Biochemistry, University of Helsinki, Viikinkaari 5 D, FIN-00140, Helsinki, Finland; [4]Department of Surgery, Helsinki University Central Hospital, Haartmaninkatu 4, FIN-00290 Helsinki, Finland; [6]Department of Pathology, Haartman Institute, Helsinki University Central Hospital, Haartmaninkatu 4, FIN-00290 Helsinki, Finland; [7]Department of Oral Diagnostics and Pathology, Institute of Dentistry, University of Oulu, FIN-90014, Oulu, Finland

**Summary** Proteolysis mediated by matrix metalloproteinases (MMPs) and serine proteinases is associated with cancer invasion and metastasis. Activation of latent proMMPs, and especially the proforms of the type IV collagen degrading gelatinases A and B (proMMP-2 and proMMP-9), is thought to be a critical step in this process. We have recently found that human tumour-associated trypsin-2 is a potent activator of proMMP-9 and it also activates proMMP-2 in vitro. Trypsinogen, MMP-2, and MMP-9 are expressed in ovarian cancer. To elucidate the function of trypsin in vivo, we studied whether high concentrations of trypsinogen-1, trypsinogen-2, their $\alpha_1$-proteinase inhibitor (API) complexes, and tumour-associated trypsin inhibitor (TATI) are associated with proMMP-2 and proMMP-9 activation in ovarian tumour cyst fluids. Zymography and immunofluorometric analysis of 61 cyst fluids showed a significant association between high trypsin concentrations and the activation of MMP-9 (*P* = 0.003–0.05). In contrast, the trypsin concentrations were inversely associated with the activation of MMP-2 (*P* = 0.01–0.02). Immunohistochemical analysis of ovarian tumour tissue demonstrated expression of trypsinogen-2 and TATI in the secretory epithelium. MMP-2 was detected both in stromal and epithelial cells whereas MMP-9 was detected in neutrophils and macrophage-like cells in stromal and epithelial areas. These results suggest that trypsin may play a role in the regulation of the MMP-dependent proteolysis associated with invasion and metastasis of ovarian cancer. © 2001 Cancer Research Campaign http://www.bjcancer.com

**Keywords**: trypsin; TATI; MMP-2; MMP-9; ovarian cancer; cyst fluid

Development of metastases is a multi-step process, and invasion of surrounding tissues by cancer cells is an initial step. The concerted action of various matrix metalloproteinases (MMPs) and serine proteinases such as plasmin and plasminogen activators is believed to be a key event in this step (Mignatti et al, 1986). We have shown that tumour-associated trypsin may participate in this process (Koivunen et al, 1991a; Sorsa et al, 1997). Two variants of the trypsinogen isoenzymes trypsinogen-1 (cationic) and trypsinogen-2 (anionic), called tumour-associated trypsinogen-1 and -2, have been shown to occur in ovarian tumour cyst fluid (Koivunen et al, 1989). Recently, extrapancreatic expression of trypsinogen has been observed in several human malignancies such as ovarian (Koivunen et al, 1989; Hirahara et al, 1995) and gastric cancer (Fujimura et al, 1998) and in cholangiocarcinoma (Terada et al, 1995). Several tumour cell lines also express trypsinogen (Koivunen et al, 1991b; Koshikawa et al, 1994). In human ovarian and gastric cancer, the trypsin expression is associated with the malignant potential of the tumours (Koivunen et al, 1990; Hirahara et al, 1998; Miyata et al, 1998; Kato et al, 1998).

*Received 31 October 2000*
*Revised 22 February 2001*
*Accepted 28 February 2001*

*Correspondence to:* A Paju

In extracellular fluids, the activity of trypsin is controlled by several inhibitors, e.g. $\alpha_1$-protease inhibitor (API), also called $\alpha_1$-antitrypsin, and $\alpha_2$-macroglobulin (Ohlsson, 1988). Tumour-associated trypsin inhibitor (TATI), which is identical to pancreatic secretory trypsin inhibitor (PSTI), is thought to prevent intracellular activation of trypsinogens (Stenman et al, 1991). TATI is expressed together with trypsinogen by several tumours and cancer cell lines (Stenman et al, 1991). TATI in serum has been used as a tumour marker for mucinous ovarian cancer and it is a prognostic factor in stage III epithelial ovarian cancer (Venesmaa et al, 1998).

MMP-2 (gelatinase A) and MMP-9 (gelatinase B) are believed to play major roles in tumour invasion by degrading type IV collagen, the main component of basement membranes (Liotta et al, 1980; Bernhard et al, 1994). It has been suggested that MMP-2 expression is a phenotypic characteristic of malignant epithelial ovarian tumours (Autio-Harmainen et al, 1993) and a potentially useful prognostic marker (Garzetti et al, 1995). Recently, Furuya et al (2000) showed that MMP-2 and MMP-9 occured at higher concentrations in cyst fluids from malignant than from benign mucinous ovarian tumours.

MMPs are frequently over-expressed in tumours (Levy et al, 1991; Davies et al, 1993) but this does not automatically imply increased proteolytic activity, because most MMPs are secreted as inactive zymogens requiring proteolytic removal of the aminoterminal domain for the expression of activity (Tryggvason et al, 1987). The urokinase type plasminogen activator (uPA) – plasmin system (Mazzieri et al, 1997) and membrane type MMP-1 (MT1-MMP, MMP-14) (Sato et al, 1994; Tokuraku et al, 1995) have been suggested to represent physiological mechanisms for the control of MMP-2 and MMP-9 activity. Recent studies have shown that human trypsin-2 is more potent than plasmin and other serine proteinases in activating several MMPs, including MMP-2 and MMP-9 (Sorsa et al, 1997), and that downregulation of trypsin-2 expression and activity in colon cancer cells is associated with decreased proMMP-9 activation (Lukkonen et al, 2000).

In the present study we investigated the involvement of trypsin in the activation of MMP-2 and MMP-9 in vivo by studying whether the concentrations of trypsins, trypsin-API-complexes, and TATI are associated with activation of proMMP-2 and proMMP-9 in ovarian tumour cyst fluid. Furthermore, we studied the immunohistochemical expression and localization of trypsinogen-2, TATI, MMP-2, and MMP-9 in ovarian tumours.

### MATERIALS AND METHODS

### Clinical specimens

Cyst fluid samples ($n$ = 61) were obtained from 58 patients (age range 13–79 years) undergoing surgery for removal of the tumour during 1986–1998 at Helsinki University Central Hospital. Of the tumours, 41 were benign and 20 malignant. The tumours were classified histologically into three main groups: mucinous and serous cystadenomas and their malignant counterparts, and other types of ovarian tumours. The benign tumours included 15 mucinous and 6 serous cystadenomas, and 20 other types of ovarian tumours (1 fibroma, 3 benign ovarian teratomas, and 16 non-neoplastic cysts). The malignant tumours included seven mucinous and nine serous cystadenocarcinomas, two mesonephroid adenocarcinomas, one mixed type carcinoma and one yolk sac tumour. The volume of cyst fluid ranged from 20 to 3000 ml. The fluids were centrifuged at 10 000 rpm for 10 min and aliquots were stored at –20˚C until analysed.

Ovarian cancer tissues were obtained from 20 patients (age range 37–84) undergoing surgery for removal of the tumour during 1990–1995 at Helsinki University Central Hospital. Samples included 10 mucinous and 10 serious ovarian cystadenocarcinomas. Tumour stage and grade were classified according to International Federation of Gynecology and Obstetrics (FIGO) (Benedet et al, 2000). Eight tumours were well-differentiated (Grade 1), seven moderately differentiated (Grade 2), and 3 poorly differentiated. For two tumours grade was not available. Nine cases represented Stage I, three Stage II, six Stage III, and two stage IV ovarian cystadenocarcinoma. This study was carried out with approval of the ethical committee of the Department of Obstetrics and Gynecology, Helsinki University Central Hospital.

### Antibodies

Monoclonal antibodies against human trypsinogen-2 (Itkonen et al, 1990) and TATI (Osman et al, 1993), and a polyclonal antibody against trypsinogen (Koivunen et al, 1989) were prepared as described. Polyclonal antibodies against MMP-2 (Turpeenniemi-Hujanen et al, 1992; Väisänen et al, 1998) and MMP-9 (Kjeldsen et al, 1993) were kindly provided by Dr Taina Turpeenniemi-Hujanen and Dr Lars Kjeldsen, respectively. The same polyclonal antibodies against MMP-2 and MMP-9 were used in Western blotting and immunohistochemistry.

### Western blotting

For Western blot analysis, 15 µl of cyst fluids (diluted 1:10 for MMP-2 and 1:5 for MMP-9 Western blotting) were mixed with a sample buffer containing 20% (v/v) glycerol, 6% (w/v) sodium dodecyl sulfate, and 0.4% (w/v) promophenol blue in 0.1 M tris-phosphate buffer, pH 6.8. The samples were run on 7.5% SDS-polyacrylamide gel electrophoresis under non-reducing conditions (Laemmli, 1970) and transferred to ProtranP Nitrocellulose Transfer Membrane (Schleicher & Schüll GmbH, Dassel, Germany) electrophoretically at 40 mA for 1 h in 25 mM Tris-HCl, pH 8.3 containing 192 mM glycine and 20% methanol. Nonspecific binding was blocked with 5% non-fat dry milk in phosphate buffered saline (PBS) (pH 7.2) for 90 min at 37 ˚C. The blots were incubated with a 1:500 dilution of polyclonal antibodies against either human trypsinogen, MMP-2, or MMP-9, or with nonimmune control serum (DAKO A/S, Glostrup, Denmark) for 3 h at 37 ˚C followed by peroxidase-conjugated second antibodies (1:200 dilution, DAKO A/S) for 1 h at 22 ˚C. After washing, the blots were developed with a solution of 60 mg/ml diaminobenzidine tetrahydrocholoride in 50 mM Tris-HCl, pH 8.0, and 0.003% $H_2O_2$ (Sorsa et al, 1997).

### Gelatin zymography

For analysis of MMP-2 and MMP-9 by zymography, cyst fluids (5 µl, diluted 1:25) were mixed with the same sample buffer as in Western blotting and run on 1.5 mm thick 7.5–10% SDS-polyacrylamide gels impregnated with 1 mg/ml gelatin (Sigma). After electrophoresis at 4 ˚C, the gels were washed for 30 min at room temperature (RT) in 50 mM Tris-HCl buffer, pH 7.8, containing 2.5% Tween-80, and 0.02% (w/v) $NaN_3$ for 30 min in the same buffer containing 1 mM $CaCl_2$ and 1 µM $ZnCl_2$. After washing, the gels were incubated in 50 mM Tris-HCl, pH 7.8, containing 150 mM NaCl, 1 mM $CaCl_2$, 1 µM $ZnCl_2$, and 0.02% $NaN_3$ for 48 h at 37 ˚C and stained with Coomassie Brilliant Blue. The total MMP content and the proportions of proforms and active forms of MMP-2 and MMP-9 were estimated with the Bio-Rad Model GS-700 Imaging Densitometer using the Molecular Analyst TM/PC program (Lukkonen et al, 2000).

### Immunofluorometric assays

Immunofluorometric assays for trypsinogen-1 and trypsinogen-2 (Itkonen et al, 1990), trypsin-1-API complex (Hedström et al, 1999), trypsin-2-API complex (Hedström et al, 1994), and TATI (Osman et al, 1993) were performed as earlier described. The detection limit was 0.1 µg/l for trypsinogen-1, 0.3 µg/l for trypsinogen-2, 0.4 µ/l for trypsin-1-API, 0.05 µg/l for trypsin-2-API, and 0.2 µg/l for TATI. The inter-and intra-assay coefficients of variation (CV) were 10–15% for trypsinogen-1, 10–12% for trypsinogen-2, 7–16% for trypsin-1-API, 4.8–6.4% and 7.3–10.4% for trypsin-2-API, and 3.0–8.6% and 13–14% for TATI (Itkonen et al, 1990; Hedström et al, 1999; Hedström et al, 1994; Osman et al, 1993).

 © 2001 Cancer Research Campaign

### Immunohistochemistry

Formalin-fixed, paraffin embedded tissue sections (4 μm) were deparaffinized in xylene and rehydrated by sequential incubation in ethanol-water solutions. The deparaffinized tissue sections were pretreated with 0.4% pepsin (Sigma), pH 1.8 for 10 min at 37˚C (MMP-2 and MMP-9), with 0.5% trypsin (Difco Laboratories, Detroit, MI, USA), pH 7.0 for 30 min at 37 ˚C (TATI), or with microwave heat in 10 mM sodium citrate buffer, pH 6.0 for 4 × 5 min (700 W) (trypsinogen-2). All sections were incubated first in 0.5% hydrogen peroxide in methanol for 15 min to quench endogenous peroxidase activity and then with non-immune mouse or rabbit serum diluted 1:67 for 15 min. The primary antibodies against trypsinogen-2 (diluted 1:1000), TATI (diluted 1:5000), MMP-2 (diluted 1:8000), and MMP-9 (diluted 1:1000) were added and the sections were incubated overnight at 4˚C. Bound antibodies were visualized by the avidin–biotin complex immunoperoxidase technique (ABC) (Elite ABC Kit, Vectastain, Vector Laboratories, Burlingame, CA, USA) following the manufacturer's instructions. The sections were incubated with the biotinylated second layer antibody and the peroxidase-labelled avidin-biotin complex for 30 min each. All dilutions were made in PBS and all incubations were carried out in humid chambers at RT. The peroxidase staining was visualized with a solution of 0.2 mg/ml 3-amino-9-ethyl-carbazole (Sigma, A-5754) in 0.05 M acetate buffer, pH 5.0, and 0.03% $H_2O_2$ for 15 min at RT. The sections were counterstained with Mayer's hemalum solution (Merck). For each tissue section, a negative control was stained by replacing the monoclonal and polyclonal primary antibodies with non-immune mouse or rabbit IgGs, respectively. Sections of specimens known to be positive for trypsinogen-2, TATI, MMP-2, and MMP-9 were used as positive controls. The effect of different pre-treatments (microwave oven treatment, incubation in 0.5% trypsin, 0.4% pepsin, or PBS) was tested for each antibody and methods resulting in optimal staining reactions were used.

### Statistical analysis

The Mann–Whitney test was performed to estimate the statistical significance of differences. All $P$ values resulted from the use of two-sided tests, and $P$ values ≤ 0.05 were considered significant.

### RESULTS

### Concentrations of trypsinogen-1, trypsinogen-2, trypsin-1-API, trypsin-2-API, and TATI

The concentrations of trypsinogen-1, trypsinogen-2, trypsin-1-API, and trypsin-2-API were higher in malignant than in benign cyst fluids ($P < 0.02$) (Figure 1) whereas there was no significant difference in TATI concentrations. In mucinous cyst fluids, the TATI concentrations were significantly higher in benign than in malignant fluids ($P < 0.04$). In serous cyst fluids trypsinogen-2, trypsin-2-API, and trypsin-1-API concentrations were higher in malignant than in benign fluids ($P < 0.02$, $P < 0.05$, and $P <0.03$, respectively) whereas there was no difference in trypsinogen-1 and TATI concentrations. The trypsinogen-2, trypsin-2-API, and TATI concentrations were higher in mucinous than in serous fluids both in malignant and benign cyst types ($P \leq 0.01$ and $P = 0.03$, respectively) (Figure 2), while there were no differences in trypsinogen-1 and trypsin-1-API concentrations. The molar ratio of trypsinogen-1 and -2 to TATI was higher in serous than in mucinous cyst fluids ($P < 0.001$).

### Activation of pro-MMP-2 and pro-MMP-9

In Western blotting and zymography, MMP-2 and MMP-9 migrated as 62 & 72 and 77 & 92 kDa bands, respectively (Figure 4A, B, D). Western blotting revealed additional 30–50 kDa fragments of both MMP-2 and MMP-9 (Figure 4A and B). In Western blotting and zymography, trypsinogen migrated as 25 & 28 kDa bands (Figure 4C and D). The MMP-9 content determined by

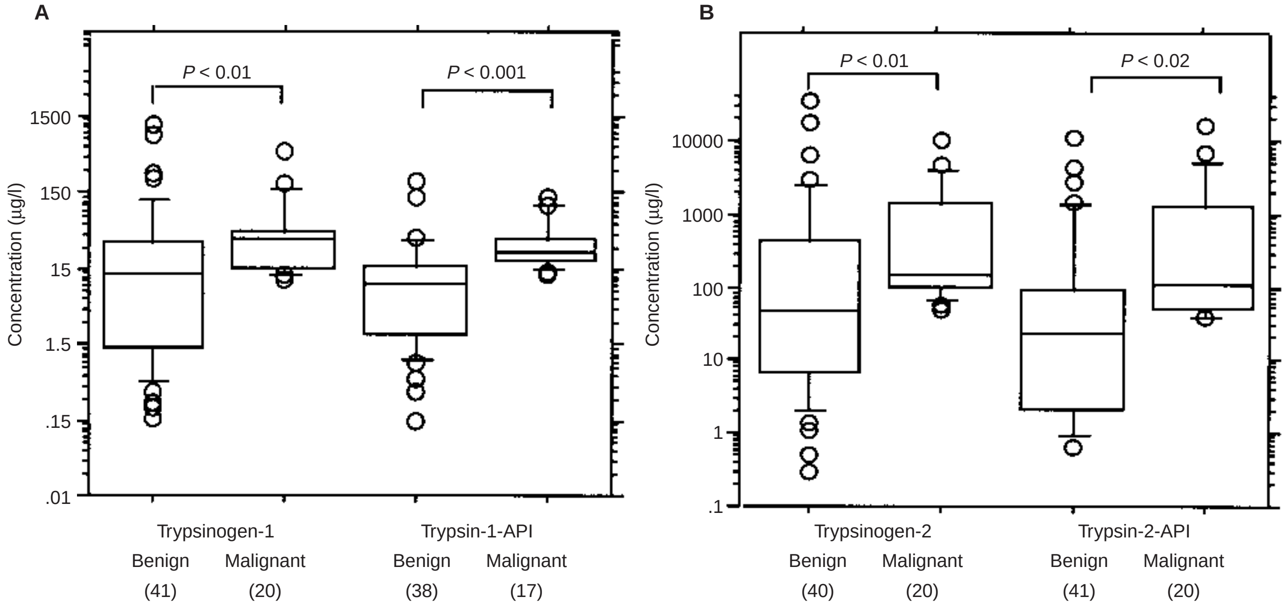

**Figure 1** Box plot of the concentrations of **(A)** trypsinogen-1 and trypsin-1-API and **(B)** trypsinogen-2 and trypsin-2-API in cyst fluids of malignant and benign ovarian tumours. The box denotes the 25th, 50th, and 75th percentiles while the whiskers represent the 10th and 90th percentiles. Values outside these limits are indicated by circles. The number of samples analysed is shown in parenthesis

*© 2001 Cancer Research Campaign*

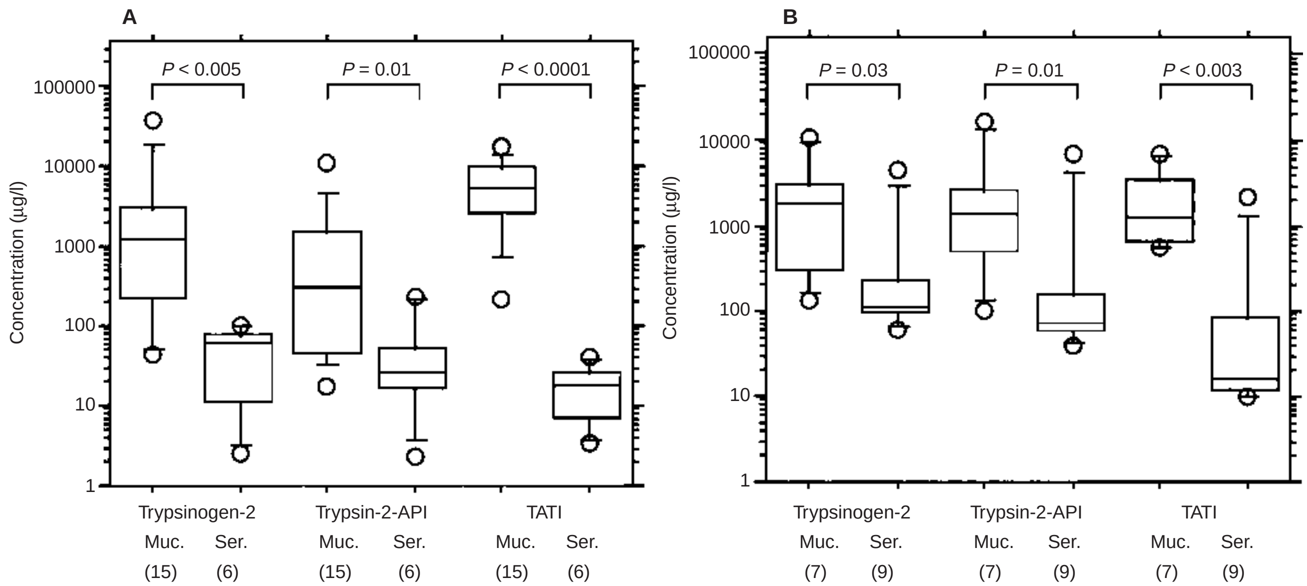

**Figure 2** Concentrations of trypsinogen-2, trypsin-2-API, and TATI in mucinous and serous cyst fluids of **(A)** benign and **(B)** malignant ovarian tumours. The symbols are as in Figure 1

zymography was significantly higher in malignant than in benign cyst fluids (2-fold, $P$ = 0.03) whereas there was no difference in MMP-2 content. The ratio of active and inactive forms of MMP-2 and MMP-9 was analysed by zymography and compared with the concentrations of trypsinogens, trypsin-API complexes, and TATI. The proportion of active MMP-9 was significantly greater in cyst fluids with high trypsinogen-2 concentration than in those with low trypsinogen-2 concentration (< 87 µg/1) ($P$ = 0.05) (Figure 4A). The proportion of active MMP-9 was also greater in cyst fluids with trypsin-1-API and trypsin-2-API concentrations higher than their median concentrations (12 and 42 µg/1, respectively) ($P$ = 0.4 and 0.1, respectively). The differences were statistically significant at cut-off levels of 30 µg/1 (trypsin-1-API) and 100 µg/1 (trypsin-2-API) ($P$ = 0.003 and 0.03, respectively).

Contrary to MMP-9, the activation of MMP-2 was inversely associated with trypsinogen-1, trypsinogen-2, trypsin-2-API, and TATI concentrations. The proportion of active form of MMP-2 was smaller when trypsinogen-1 and trypsin-2-API concentrations were higher than their median concentrations (20 and 42 µg/l, respectively) ($P$ = 0.02). Similarly, less active MMP-2 occurred in cyst fluids with trypsinogen-2 and TATI concentrations higher than their median concentrations (87 and 25 µg/l, respectively) ($P$ = 0.1 and 0.2, respectively). The results were statistically significant when cut-off concentrations 50 µg/l (trypsinogen-2) and 1000 µg/l (TATI) were used ($P$ = 0.01 and 0.04, respectively) (Figure 4B).

### Localization of trypsinogen and MMP immunoreactivity in ovarian cancer tissues

By immunohistochemistry, trypsinogen-2 was found in the epithelium of 8 of 10 (80%) mucinous and in 4 of 10 (40%) serous cystadenocarcinomas. TATI was detected in the epithelium of 9 of 10 (90%) mucinous and in 1 of 10 (10%) serous cystadenocarcinomas (Table 1, Figure 5). Of mucinous cystadenocarcinomas 9 of 10 (90%) showed MMP-2 immunoreactivity in stromal cells, 5 of 10 (50%) in epithelium, and all (100%) in vascular endothelium. Of serous cystadenocarcinomas 8 of 10 (80%) showed MMP-2 immunoreactivity in stromal cells, 4 of 10 (40%) in epithelium, and 6 of 10 (60%) in vascular endothelium. MMP-9-positive neutrophils and monocyte-macrophage-like cells were detected in stromal and epithelial areas in 8 of 10 (80%) mucinous and 7 of 10 (70%) serous cystadenocarcinomas (Table 1, Figure 6).

### DISCUSSION

Tumour-associated trypsinogens and TATI are produced by various human cancers (Koivunen et al, 1989; Hirahara et al,

**Table 1** Summary of trypsinogen-2, TATI, MMP-2, and MMP-9 immunostaining of ovarian cancer specimens

| | Trypsinogen-2 (0/1/2–3) | | TATI (0/1/2–3) | MMP-2 (0/1/2–3) | | | MMP-9 (+/–) |
|---|---|---|---|---|---|---|---|
| Histology | n | (EP) | (EP) | (S) | (EP) | (EN) | (M/N) |
| Mucinous | 10 | 2/2/6 | 1/1/8 | 1/5/4 | 5/3/2 | 0/7/3 | 8/2 |
| Serous | 10 | 6/2/2 | 9/0/1 | 2/5/3 | 6/2/2 | 4/2/4 | 7/3 |

0 = immunoreactivity absent; 1 = weak immunoreactivity; 2–3 = moderate or strong immunoreactivity; +/−= immunoreactive cells detected/not detected. EP = epithelium; S = stroma; EN = endothelium; M/N = macrophages and/or neutrophils; TATI = tumour-associated trypsin inhibitor; MMP = matrix metalloproteinase.

 *© 2001 Cancer Research Campaign*

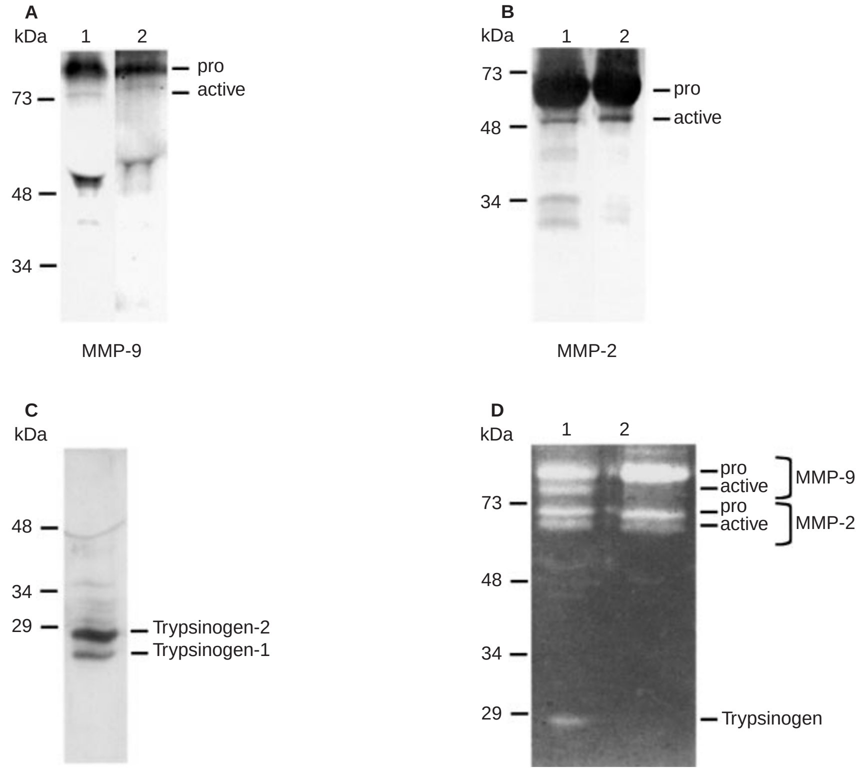

**Figure 3** Western blot analysis of cyst fluids from two patients with benign ovarian cysts using a polyclonal antibody against MMP-9 **(A)** and a polyclonal antibody against MMP-2 **(B)**. Panel **(C)** shows Western blot analysis of cyst fluid from a patient with mucinous ovarian tumour using a polyclonal antibody against trypsinogen. Panel **(D)** shows zymography of cyst fluids from two patients with mucinous ovarian tumour

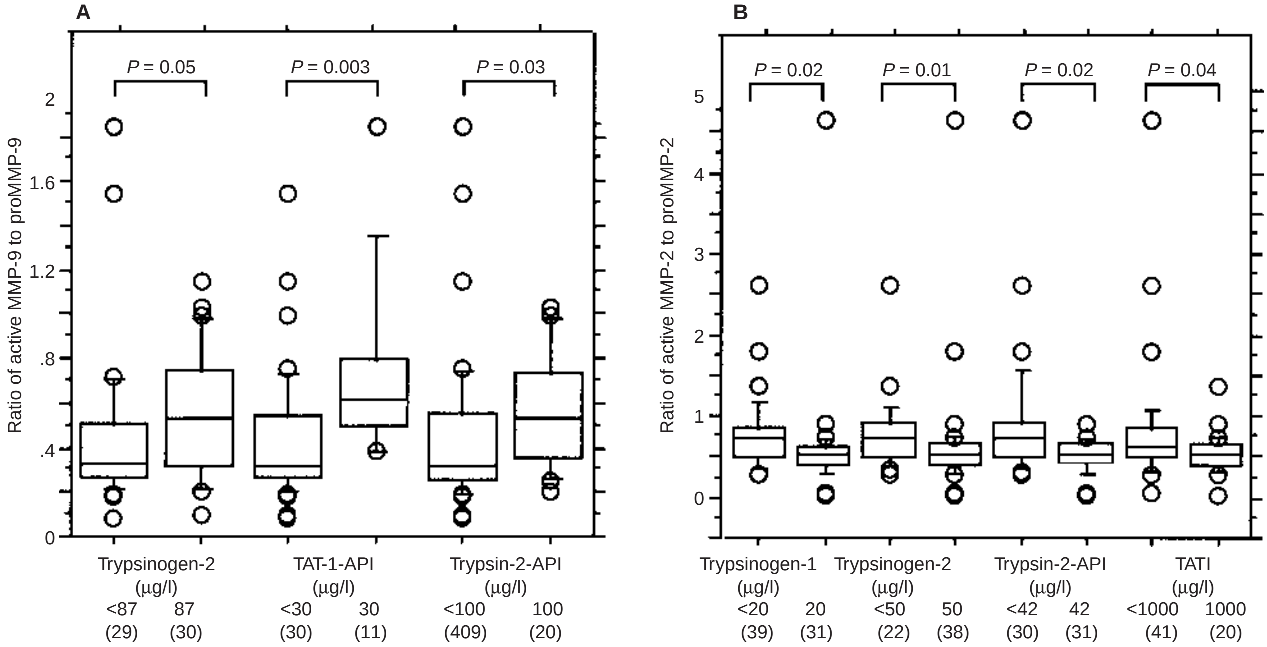

**Figure 4 (A)** Ratio of active MMP-9 to proMMP-9 in ovarian tumour cyst fluids in relation to trypsinogen-2, trypsin-1-API, and trypsin-2-API concentrations. **(B)** Ratio of active MMP-2 to proMMP-2 in ovarian tumour cyst fluids with respect to trypsinogen-1, trypsinogen-2, trypsin-2-API, and TATI concentration. The symbols are as in Figure 1

*© 2001 Cancer Research Campaign*

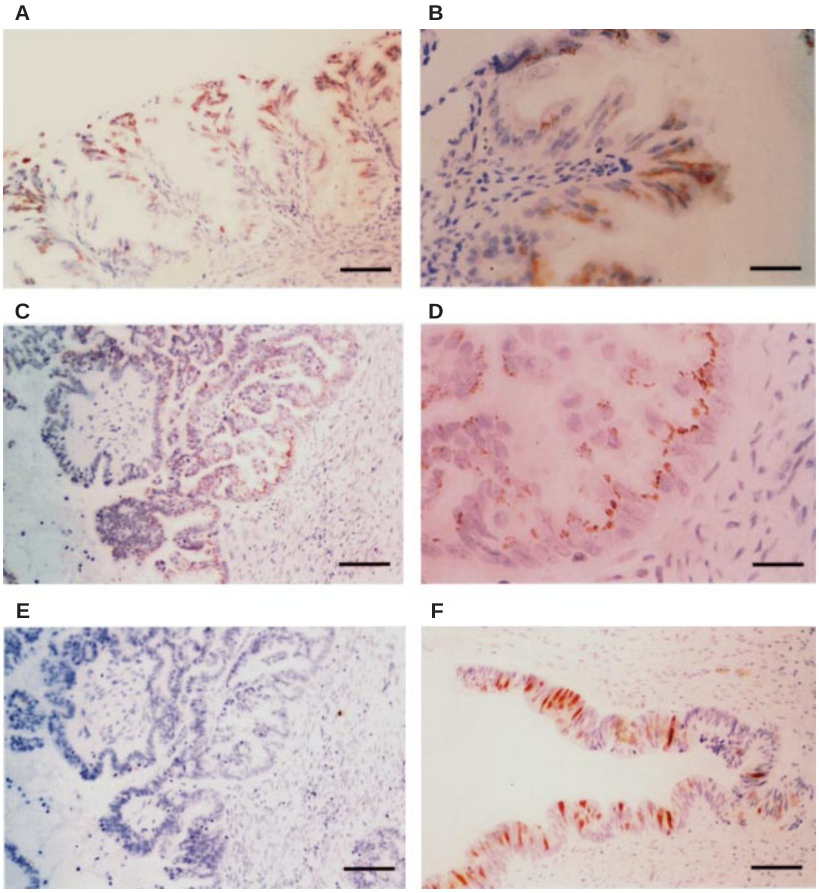

**Figure 5** Immunohistochemical expression of trypsinogen-2 in a grade 1 mucinous cystadenocarcinoma **(A, B)**, trypsinogen-2 in a grade 1 serous cystadenocarcinoma **(C, D)**, and tumour-associated trypsin inhibitor in a grade 2 mucinous cystadenocarcinoma **(F)**. Panel **(E)** shows a negative control. Scale bars, 50 μm **(A, C, E, F)** and 12.5 μm **(B, D)**

1995; Terada et al, 1995) and tumour cell lines (Koivunen et al, 1991b; Koshikawa et al, 1994). The main tumour-associated isoenzyme, trypsin-2, activates pro-urokinase-type plasminogen activator (pro-uPA) (Koivunen et al, 1989) and in vitro it is the most efficient activator of the 92 kDa gelatinase B (MMP-9) known thus far (Sorsa et al, 1997). Therefore, trypsin may participate in cancer cell-mediated proteolysis by directly degrading the extracellular matrix or by activating other proteinases (Koivunen et al, 1991a).

The expression of MMP-2 and MMP-9 correlates with the invasive and metastatic potential of various tumours (Höyhtyä et al, 1990; Juarez et al, 1993; Bernhard et al, 1994). The zymogen forms of MMP-2 and MMP-9 can be activated by several proteinases in vitro and it has been suggested that cell surface-bound MMP-14 plays an important role in the activation of MMP-2 (Sato et al, 1994) and plasmin in the activation of MMP-9 (Mazzieri et al, 1997). Most studies concerning the activation of MMPs have been performed in vitro, and only a few studies have provided evidence of activation in vivo. A correlation between MMP-14 expression and activation of MMP-2 has been observed in cancer tissues (Tokuraku et al, 1995) suggesting that MMP-14 is a physiological activator of MMP-2. In an in vivo model of acute lung injury neutrophil elastase was found to be a potential physiological activator of MMP-9 (Ferry et al, 1997).

The results of the present study show that high concentrations of trypsinogens and trypsin-API complexes in cyst fluids of ovarian tumours correlate with increased MMP-9 and decreased MMP-2 activation. This suggests that trypsin-1 and trypsin-2 may be involved in the activation of MMP-9 but not MMP-2 in vivo. Although human trypsin-2 reportedly activates MMP-2 in vitro it has been observed that MMP-2 is further fragmented in its C-terminal region during a prolonged incubation with trypsin-2 (Sorsa et al, 1997). It is possible that trypsin, a very efficient proteinase, may inactivate MMP-2 or its activator MMP-14 when occurring at high concentrations. Several other factors, e.g. other MMPs, the uPA-plasmin system, and tissue inhibitors of matrix metalloproteinases (TIMPs) also affect the activity of MMP-2 and MMP-9. TIMP-1 which is present in cyst fluids at high concentrations (Furuya et al, 2000) can reduce but not abolish the activation of MMP-9 by trypsin-2 (Sorsa et al, 1997).

In line with the findings of Furuya et al (2000), the total MMP-9 content was significantly higher in malignant than in benign cyst fluids whereas there was no difference in the total MMP-2 content. Consistent with the study of Koivunen et al (1990), we found that the concentrations of trypsinogen-1 and trypsinogen-2, but not those of TATI were significantly higher in malignant than in benign cyst fluids. In addition, the concentrations of trypsin-API complexes, which reflect the proportion of active trypsin, were significantly higher in malignant than in benign fluids. Interestingly, the molar ratio of trypsinogen to TATI was significantly higher in serous than in mucinous cyst fluids. This may be related to the poorer prognosis of serous than mucinous

 *© 2001 Cancer Research Campaign*

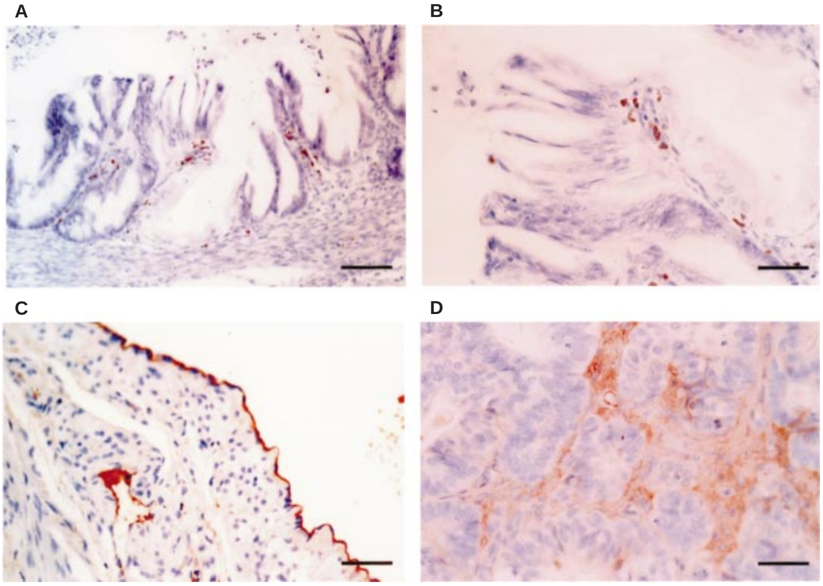

**Figure 6** Immunohistochemical expression of MMP-9 in neutrophils and macrophages in a grade 1 mucinous cystadenocarcinoma **(A, B)**, MMP-2 in epithelial and endothelial cells of grade 2 serous cystadenocarcinoma **(C)**, and MMP-2 in stromal cells of grade 2 serous cystadenocarcinoma **(D)**. For each tissue section control stainings were negative. Scale bars, 50 µm **(A, C)** and 25 µm **(B, D)**

ovarian carcinomas at an early stage of the disease (Vergote et al, 1993).

By immunohistochemistry, we detected trypsinogen-2 and TATI in the secretory epithelium of ovarian carcinomas as has been earlier described (Ueda et al, 1989; Hirahara et al, 1995). Trypsinogen-2 and TATI immunoreactivity was detected more frequently in mucinous than in serous tumours and in agreement with this, the trypsinogen and TATI concentrations were found to be significantly higher in mucinous than in serous cyst fluids. In ovarian carcinomas, MMP-2 mRNA expression has been detected mainly in stromal fibroblasts and endothelial cells but only rarely in epithelial cells (Autio-Harmainen et al, 1993; Afzal et al, 1998; Naylor et al, 1994) whereas immunoreactive MMP-2 has frequently been detected in the cytoplasm and at the surface of epithelial tumor cells (Autio-Harmainen et al, 1993; Afzal et al, 1996; De Nictolis et al, 1996; Höyhtyä et al, 1994). However, several cultured ovarian carcinoma cells produce MMP-2 and MMP-9 (Moser et al, 1994; Fishman et al, 1997; Boyd and Balkwill, 1999; Westerlund et al, 1997) and a cell-cell interaction in a coculture of fibroblasts and carcinoma cells stimulates MMP-2 expression and *in vitro* cancer cell invasion (Boyd and Blakwill, 1999; Westerlund et al, 1997). In agreement with earlier studies (Autio-Harmainen et al, 1993; Afzal et al, 1996; De Nictolis et al, 1996; Höyhtyä et al, 1994) we found by immunohistochemistry that MMP-2 localized to the vascular endothelial, epithelial, and stromal cells. Furthermore, in line with several studies suggesting stromal macrophages and tumour-infiltrating neutrophils as major sources of MMP-9 in invasive cancers, e.g. in colon (Nielsen et al, 1996; Pyke et al, 1993), breast (Davies et al, 1993a), and bladder cancer (Davies et al, 1993b) and in ovarian carcinoma (Naylor et al, 1994), we detected MMP-9 immunoreactivity in neutrophils and monocyte-macrophage-like cells both in stromal and epithelial areas. This is in contrast to the findings of Huang et al (2000), who recently demonstrated MMP-9 mRNA and protein both in stromal and neoplastic cells of ovarian carcinomas (Huang et al, 2000). The immunohistochemical findings support the notion that stromal cells, inflammatory cells, and cancer cells cooperatively produce, induce, and activate tissue-destructive serine proteinases and MMPs that facilitate tumour invasion and metastatic spread (Pyke et al, 1993).

In conclusion, high levels of trypsin-1 and -2 in ovarian tumour cyst fluids were associated with increased activation of MMP-9 but decreased activation of MMP-2. These findings suggest that trypsin may represent a physiological regulator of the matrix metalloproteinase cascade involved in local invasion and metastatic spread of ovarian tumours.

### ACKNOWLEDGEMENTS

We thank Mrs Anne Ahmanheimo, Ms Maarit Leinimaa, Mrs Elina Laitinen, and Mrs Sari Nieminen for their excellent technical assistance. This work was supported by grants from the Finnish Cancer Foundation, the Maud Kuistila Foundation, the Sigrid Juselius Foundation, the Finnish Academy of Sciences, Helsinki University Central Hospital, University of Helsinki, and the Wilhelm Stockmann Foundation.

### REFERENCES

Afzal S, Lalani E-N, Foulkes WD, Boyce B, Tickle S, Cardillo MR, Baker T, Pignatelli M and Stamp GW (1996) Matrix metalloproteinase-2 and tissue inhibitor of metalloproteinase-2 expression and synthetic matrix metalloproteinase-2 inhibitor binding in ovarian carcinomas and tumor cell lines. *Lab Invest* **74**: 406–421

Afzal S, Lalani E-N, Poulsom R, Stubbs A, Rowlingson G, Sato H, Seiki M and Stamp GWH (1998) MT1-MMP and MMP-2 mRNA expression in human ovarian tumors: possible implications for the role of desmoplastic fibroblasts. *Hum Pathol* **29**: 155–165

Autio-Harmainen H, Karttunen T, Hurskainen T, Höyhtyä M, Kauppila A and Tryggvason K (1993) Expression of 72 kilodalton type IV collagenase (gelatinase A) in benign and malignant ovarian tumors. *Lab Invest* **69**: 312–321

Benedet JL, Bender H, Jones III H, Ngan HYS and Pecorelli S (2000) FIGO staging classifications and clinical practice guidelines in the management of gynecologic cancers. *Int J Gynecol Obstet* **70**: 209–262

Bernhard EJ, Gruber SB and Muschel RJ (1994) Direct evidence linking expression of matrix metalloproteinase 9 (92-kDa gelatinase / collagenase) to the metastatic phenotype in transformed rat embryo cells. *Proc Natl Acad Sci USA* **91**: 4293–4297

Boyd RS and Balkwill FR (1999) MMP-2 release and activation in ovarian carcinoma: the role of fibroblasts. *Br J Cancer* **80**: 315–321

Davies B, Miles DW, Happerfield LC, Naylor MS, Bobrow LG, Rubens RD and Balkwill FR (1993a) Activity of type IV collagenases in benign and malignant breast disease. *Br J Cancer* **67**: 1126–1131

Davies B, Waxman J, Wasan H, Abel P, Williams G, Krausz T, Neal D, Thomas D, Hanby A and Balkwill F (1993b) Levels of metalloproteinases in bladder cancer correlate with tumor grade and invasion. *Cancer Res* **53**: 5365–5369

De Nictolis M, Garbisa S, Lucarini G, Goteri G, Masiero L, Ciavattini A, Garzetti GG, Stetler-Stevenson WG, Fabris G, Biagini G and Prat J (1996) 72–kilodalton type IV collagenase, type IV collagen, and Ki 67 antigen in serous tumors or the ovary: a clinicopathologic, immunohistochemical, and Serological study. *Int J Gynecol Pathol* **15**: 102–109

Ferry G, Lonchampt M, Pennel L, de Nanteuil G, Canet E and Tucker GC (1997) Activation of MMP-9 by neutrophil elastase in an in vivo model of acute lung injury. *FEBS Lett* **402**: 111–115

Fishman D, Bafetti LM, Banionis S, Kearns AS, Chilukuri K and Stack MS (1997) Production of extracellular matrix-degrading proteinases of primary cultures of human epithelial ovarian carcinoma cells. *Cancer* **80**: 1457–1463

Fujimura T, Ohta T, Kitagawa H, Fushida S, Nishimura G-I, Yonemura Y, Elnemr A, Miwa K and Nakanuma Y (1998) Trypsinogen expression and early detection for peritoneal dissemination in gastric cancer. *J Surg Oncol* **69**: 71–75

Furuya M, Ishikura H, Kawarada Y, Ogawa Y, Sakuragi N, Fujimoto S and Yoshiki T (2000) Expression of matrix metalloproteinases and related tissue inhibitors in the cyst fluids of ovarian mucinous neoplasms. *Gynecol Oncol* **78**: 106–112,doi: 10.1006 / gyno.2000.5856

Garzetti GG, Ciavattini A, Lucarini G, Goteri G, de Nictolis M, Garbisa S, Masiero L, Romanini C and Graziella B (1995) Tissue and serum metalloproteinase (MMP-2) expression in advanced ovarian serous cystoadenocarcinomas: clinical and prognostic implications. *Anticancer Res* **15**: 2799–2804

Hedström J, Leinonen J, Sainio V and Stenman U-H (1994) Time-resolved immunofluorometric assay of trypsin-2 complexed with a-1-antitrypsin in serum. *Clin Chem* **40**: 1761–1765

Hedström J, Haglund C, Kemppainen E, Leinimaa M, Leinonen J and Stenman U-H (1999) Time-resolved immunofluorometric assay of trypsin-1 complexed with $\alpha_1$-antitrypsin in serum: increased immunoreactivity in patients with biliary tract cancer. *Clin Chem* **45**: 1768–1773

Hirahara F, Miyagi Y, Miyagi E, Yasumitsu H, Koshikawa N, Nagashima Y, Kitamura H, Minaguchi H, Umeda M and Miyazaki K (1995) Trypsinogen expression in human ovarian carcinomas. *Int J Cancer* **63**: 176–181

Hirahara F, Miyagi E, Nagashima Y, Miyagi Y, Yasumitsu H, Koshikawa N, Nakatani Y, Nakazawa T, Udagawa K, Kitamura H, Minaguchi H and Miyazaki K (1998) Differential expression of trypsin in human ovarian carcinomas and low-malignant-potential tumors. *Gynecol Oncol* **68**: 162–165

Höyhtyä M, Hujanen E, Turpeenniemi-Hujanen T, Thorgeirsson U, Liotta LA and Tryggvason K (1990) Modulation of type-IV collagenase activity and invasive behavior of metastatic human melanoma (A2058) cells in vitro by monoclonal antibodies to type-IV collagenase. *Int J Cancer* **46**: 282–286

Höyhtyä M, Fridman R, Komarek D, Porter JK, Stetler-Stevenson WG, Liotta LA and Liang CM (1994) Immunohistochemical localization of matrix metalloproteinase 2 and its specific inhibitor TIMP-2 in neoplastic tissues with monoclonal antibodies. *Int J Cancer* **56**: 500–505

Huang LW, Garrett AP, Bell DA, Welch WR, Berkowitz RS and Mok SC (2000) Differential expression of matrix metalloproteinase-9 and tissue inhibitor of metalloproteinase-1 protein and mRNA in epithelial ovarian tumors. *Gynecol Oncol* **77**: 369–376

Itkonen O, Koivunen E, Hurme M, Alfthan H, Schröder T and Stenman U-H (1990) Time-resolved immunofluorometric assays for trypsinogen-1 and 2 in serum reveal preferential elevation of trypsinogen-2 in pancreatitis. *J Lab Clin Med* **115**: 712–718

Juarez J, Clayman G, Nakajima M, Tanabe KK, Saya H, Nicolson GL and Boyd D (1993) Role and regulation of expression of 92-kDa type-IV collagenase (MMP-9) in 2 invasive squamous-cell-carcinoma cell lines of the oral cavity. *Int J Cancer* **55**: 10–18

Kato Y, Nagashima Y, Koshikawa N, Miyagi Y, Yasumitsu H and Miyazaki K (1998) Production of trypsins by human gastric cancer cells correlates with their malignant phenotype. *Eur J Cancer* **34**: 1117–1123

Kjeldsen L, Johnsen AH, Sengelov H and Borregaard N (1993) Isolation and primary structure of NGAL, a novel protein associated with human neutrophil gelatinase. *J Biol Chem* **268**: 10425–10432

Koivunen E, Huhtala M-L and Stenman U-H (1989) Human ovarian tumor-associated trypsin. Its purification and characterization from mucinous cyst fluid and identification as an activator of pro-urokinase. *J Biol Chem* **264**: 14095–14099

Koivunen E, Itkonen O, Halila H and Stenman U-H (1990) Cyst fluid of ovarian cancer patients contains high concentrations of trypsinogen-2. *Cancer Res* **50**: 2375–2378

Koivunen E, Ristimäki A, Itkonen O, Osman S, Vuento M and Stenman U-H (1991a) Tumor-associated trypsin participates in cancer cell-mediated degradation of extracellular matrix. *Cancer Res* **51**: 2107–2112

Koivunen E, Saksela O, Itkonen O, Osman S, Huhtala M-L and Stenman U-H (1991b) Human colon carcinoma, fibrosarcoma and leukemia cell lines produce tumor-associated trypsinogen. *Int J Cancer* **47**: 592–596

Koshikawa N, Yasumitsu H, Nagashima Y, Umeda M and Miyazaki K (1994) Identification of one-and two-chain forms of trypsinogen 1 produced by a human gastric adenocarcinoma cell line. *Biochem J* **303**: 187–190

Laemmli UK (1970) Cleavage of structural proteins during the assembly of the head of bacteriophage T4. *Nature* **227**: 680–685

Levy AT, Cioce V, Sobel ME, Garbisa S, Grigioni WF, Liotta LA and Stetler-Stevenson, SW (1991) Increased expression of the Mr 72,000 type IV collagenase in human colonic adenocarcinoma. *Cancer Res* **51**: 439–444

Liotta LA, Tryggvason K, Garbisa S, Hart I, Foltz CM and Shafie S (1980) Metastatic potential correlates with enzymatic degradation of basement membrane collagen. *Nature* **284**: 67–68

Lukkonen A, Sorsa T, Salo T, Tervahartiala T, Koivunen E, Golub L, Simon S and Stenman U-H (2000) Down-regulation of trypsinogen-2 expression by chemically modified tetracyclines: association with reduced cancer cell migration. *Int J Cancer* **86**: 577–581

Mazzieri R, Masiero L, Zanetta L, Monea S, Onisto M, Garbisa S and Mignatti P (1997) Control of type IV collagenase activity by components of the urokinase-plasmin system: a regulatory mechanism with cell-bound reactants. *EMBO J* **16**: 2319–2332

Mignatti P, Robbins E and Rifkin DB (1986) Tumor invasion through the human amniotic membrane: requirement for a proteinase cascade. *Cell* **47**: 487–498

Miyata S, Miyagi Y, Koshikawa N, Nagashima Y, Kato Y, Yasumitsu H, Hirahara F, Misugi K and Miyazaki K (1998) Stimulation of cellular growth and adhesion to fibronectin and vitronectin in culture and tumorigenicity in nude mice by overexpression of trypsinogen in human gastric cancer cells. *Clin Exp Metastasis* **16**: 613–622

Moser TL, Young TN, Rodriguez GC, Pizzo SV, Bast RC and Stack MS (1994) Secretion of extracellular matrix-degrading proteinases is increased in epithelial ovarian carcinoma. *Int J Cancer* **56**: 552–559

Naylor MS, Stamp GW, Davies BD and Balkwill FR (1994) Expression and activity of MMPS and their regulators in ovarian cancer. *Int J Cancer* **58**: 50–56

Nielsen BS, Timshel S, Kjeldsen L, Sehested M, Pyke C, Borregaard N and Dano K (1996) 92 kDa type IV collagenase (MMP-9) is expressed in neutrophils and macrophages but not in malignant epithelial cells in human colon cancer. *Int J Cancer* **65**: 57–62

Ohlsson K (1988) Acute pancreatitis. Biochemical, pathophysiological and therapeutical aspects. *Acta Gastroenterol Belg* **51**: 3–12

Osman S, Turpeinen U, Itkonen O and Stenman U-H (1993) Optimization of a time-resolved immunofluorometric assay for tumor-associated trypsin inhibitor (TATI) using the streptavidin-biotin system. *J Immunol Methods* **161**: 97–106

Pyke C, Ralfkiær E, Tryggvason K and Danø K (1993) Messenger RNA for two type IV collagenases is located in stromal cells in human colon cancer. *Am J Pathol* **142**: 359–365

Sato H, Takino T, Okada Y, Cao J, Shinagawa A, Yamamoto E and Seiki M (1994) A matrix metalloproteinase expressed on the surface of invasive tumour cells. *Nature* **370**: 61–65

Sorsa T, Salo T, Koivunen E, Tyynelä J, Konttinen YT, Bergmann U, Tuuttila A, Niemi E, Teronen O, Heikkilä P, Tschesche H, Leinonen J, Osman S and Stenman U-H (1997) Activation of type IV procollagenases by human tumor-associated trypsin-2. *J Biol Chem* **272**: 21067–21074

Stenman U-H, Koivunen E and Itkonen O (1991) Biology and function of tumor-associated trypsin inhibitor, TATI. *Scand J Clin Lab Invest Suppl* **207**: 5–9

Terada T, Ohta T, Minato H and Nakanuma Y (1995) Expression of pancreatic trypsinogen/trypsin and cathepsin B in human cholangiocarcinomas and hepatocellular carcinomas. *Hum Pathol* **26**: 746–752

Tokuraku M, Sato H, Murakami S, Okada Y, Watanabe Y and Seiki M (1995) Activation of the precursor of gelatinase A/72 kDa type IV collagenase/MMP-2 in lung carcinomas correlate with the expression of membrane-type matrix metalloproteinase (MT-MMP) and with lymph node metastasis. *Int J Cancer* **64**: 355–359

 *© 2001 Cancer Research Campaign*

Tryggvason K, Höyhtyä M and Salo T (1987) Proteolytic degradation of extracellular matrix in tumor invasion. *Biochim Biophys Acta* **907**: 191–217

Turpeenniemi-Hujanen T, Ronnberg L, Kauppila A and Puistola U (1992) Laminin in the human embryo implantation: analogy to the invasion by malignant cells. *Fertil Steril* **58**: 105–13

Ueda G, Shimizu C, Tanaka Y, Inoue M, Tanizawa O, Ogawa M and Mori T (1989) Immunohistochemical demonstration of pancreatic secretory trypsin inhibitor in gynecologic tumors. *Gynecol Oncol* **32**: 37–40

Väisänen A, Kallioinen M, Taskinen PJ and Turpeenniemi-Hujanen T (1998)Prognostic value of MMP-2 immunoreactive protein (72 kD type IV collagenase) in primary skin melanoma. *J Pathol* **186**: 51–58

Venesmaa P, Stenman U-H, Forss M, Leminen A, Lehtovirta P, Vartiainen J and Paavonen J (1998) Pre-operative serum level of tumour-associated trypsin inhibitor and residual tumor size as prognostic indicators in Stage III epithelial ovarian cancer. *Br J Obstet Gynaecol* **105**: 508–511

Vergote IB, Kærn J, Abeler VM, Pettersen EO, De Vos LN and Tropé CG (1993) Analysis of prognostic factors in stage I epithelial ovarian carcinoma: importance of degree of differentiation and deoxyribonucleic acid ploidy in predicting relapse. *Am J Obstet Gynecol* **169**: 40–52

Westerlund A, Hujanen E, Puistola U and Turpeenniemi-Hujanen T (1997) Fibroblasts stimulate human ovarian cancer cell invasion and expression of 72-kDa gelatinase A (MMP-2). *Gynecol Oncol* **67**: 76–82

*© 2001 Cancer Research Campaign*
